# A Novel Manufacturing Concept of LCP Fiber-Reinforced GPET-Based Sandwich Structures with an FDM 3D-Printed Core

**DOI:** 10.3390/ma15155405

**Published:** 2022-08-05

**Authors:** Jacek Andrzejewski, Marcin Gronikowski, Joanna Aniśko

**Affiliations:** 1 Faculty of Mechanical Engineering, Institute of Materials Technology, Poznan University of Technology, ul. Piotrowo 3, 61-138 Poznan, Poland; 2 MATRIX Students Club, Poznan University of Technology, ul. Piotrowo 3, 61-138 Poznan, Poland; 3 Faculty of Materials Engineering and Technical Physics, Poznan University of Technology, ul. Piotrowo 3, 60-965 Poznan, Poland

**Keywords:** FDM printing, hybrid manufacturing, sandwich composite structure, fiber reinforcement, impact resistance

## Abstract

The presented research was focused on the development of a new method of sandwich structure manufacturing involving FDM-printing (fused deposition modeling) techniques and compression molding. The presented concept allows for the preparation of thermoplastic-based composites with enhanced mechanical properties. The sample preparation process consists of 3D printing the sandwich’s core structure using the FDM method. For comparison purposes, we used two types of GPET (copolymer of polyethylene terephthalate)-based filaments, pure resin, and carbon fiber (CF)-reinforced filaments. The outer reinforcing layer “skins” of the sandwich structure were prepared from the compression molded prepregs made from the LCP (liquid-crystal polymer)-fiber fabric with the GPET-based matrix. The final product consisting of an FDM-printed core and LCP-based prepreg was prepared using the compression molding method. The prepared samples were subjected to detailed materials analyses, including thermal analyses (thermogravimetry-TGA, differencial scanning calorimetry-DSC, and dynamic thermal-mechanical analysis-DMTA) and mechanical tests (tensile, flexural, and impact). As indicated by the static test results, the modulus and strength of the prepared composites were slightly improved; however, the stiffness of the prepared materials was more related to the presence of the CF-reinforced filament than the presence of the composite prepreg. The main advantage of using the developed method is revealed during impact tests. Due to the presence of long LCP fibers, the prepared sandwich samples are characterized by very high impact resistance. The impact strength increased from 1.7 kJ/m^2^ for pure GPET samples to 50.4 kJ/m^2^ for sandwich composites. For GPET/CF samples, the increase is even greater. The advantages of the developed solution were illustrated during puncture tests in which none of the sandwich samples were pierced.

## 1. Introduction

For many years, the FDM technique (fused deposition modeling) has been the primary 3D-printing method, although it is neither the fastest nor the most accurate. The popularity of this method was determined primarily by the low price of the devices (printers), the availability of a wide range of processed materials, and the possibility of their easy replacement [1,2,3]. The most popular plastics used in this method are poly (lactic acid)-PLA [4,5,6], acrylonitrile-butadiene-styrene copolymer-ABS [7,8,9], and polyethylene terephthalate glycol-modified copolymer-GPET [10,11]. The popularity of PLA is mainly related to the excellent processability of this material; the low melting point and low processing shrinkage render this material perfect for FDM process conditions. Unfortunately, despite the high accuracy of the 3D model shape, the parts prepared with the use of PLA are characterized by high brittleness and low thermal resistance (≈60 °C), which limits their use in technical applications [5,12,13]. In the case of more demanding applications, the more popular material is the ABS copolymer; in this case, the range of applications reaches even 100 °C, and the impact strength is also significantly improved [14,15]. Unfortunately, due to the more difficult processability of the material, the accurate mapping of the designed model geometry is much more difficult for ABS. It often requires the use of more advanced printing machines. The third-most popular material is GPET; as an amorphous polyethylene terephthalate copolymer, it has similar properties to PLA, but due to its higher glass transition temperature, its maximum application temperature reaches about 70 °C.

All popular materials intended for FDM printing are also available as filled or reinforced filaments. Most often, they are glass or carbon fibers [16,17,18], although there are also commercial materials based on nanoadditives such as carbon nanotubes or graphene [19,20,21]. Unfortunately, due to the characteristics of the printing process, the content of these additives rarely exceeds 20%. A higher filler content increases the viscosity of the polymer, which leads to a significant flow resistance during the flow through the forming die. It is worth mentioning that the FDM melting process is not supported by the screw unit system, which limits the effectiveness of material plasticization. In selected cases, the use of screw plasticizing systems occurs only for large-size machines with the required high flow rate [22,23,24,25]. Therefore, the traditional FDM method cannot process composite materials with a high degree of filler content; therefore, new methods are constantly being developed to increase the effectiveness of the reinforcement.

One of such solutions, intensively developed in recent years, is the composite filament co-extrusion method (CFC-FDM), in which the printed model is reinforced by a bundle of continuous fibers introduced into the polymer melt [26,27,28,29]. This method requires the use of a modified print head equipped with a separate nozzle for supplying reinforcing fibers. Currently, there are several commercial systems available on the market that enable printing with this method, and the most popular CFC-FDM printers are produced by “Markforged” and “Anisoprint” companies, but due to the need to pre-coat reinforcing fibers and the use of dedicated polymer materials, the production costs associated with this method are still relatively high.

The low efficiency of short reinforcing fibers concerns not only 3D-printing techniques. Similar problems occur in the case of injection molding technology, where the concept of using LFT (long fiber thermoplastic)-type granules allows an increase in the effectiveness of the reinforcement. However, it still does not come close to the results obtained by polymer laminates [30,31,32]. Therefore, one of the latest trends in overmolding technologies is the use of composite prepregs as inserts placed inside the mold’s cavity [33,34,35,36,37]. After joining the prepreg with the injection molded material, the properties of the obtained part combine the strength characteristic of reinforced laminates and the possibility of shaping the complex geometries that characterize the injection molding technique. The concept of the presented works assumes obtaining a similar type of hybrid materials using the 3D-printing method. Similarly to the overmolding process, the laminated prepreg will be permanently attached to the printed element using the compression-molding method additionally so that samples with a sandwich structure can be obtained. In the case of the presented research, the main objective will be to assess the quality of the joint surface formed during the preparation of the sandwich structure for materials based on the GPET copolymer.

The literature review shows that solutions of this type have not been used commercially so far; however, there are examples of scientific research on a similar subject. Sandwich structures can be made entirely in the FDM technique [38,39,40,41]. Still, the research so far shows that despite the possibility of shaping the composite core in a very wide range, the improvement in the properties of this type of material is relatively small. The aforementioned continuous-filament printing technique CFC-FDM was used to prepare sandwich samples. The research conducted by Sugiyama et al. confirmed the possibility of using 3D printing for the production of this type of structures [42]. However, the obtained results do not show a significant advantage of the CFC-FDM technique. A different concept of the work assumes using a ready-made fabric reinforced prepreg and combining it with a 3D-printed composite core using the adhesive technique [43,44,45]. The advantage of this solution is the possibility of using a wide range of printing methods, including printing photocurable materials or thermosets.

In most of the already-developed solutions, the structure of the molded part was reinforced by a prepreg with the addition of glass or carbon fibers. Considering the high fiber content in this type of laminate, the apparent fact is the increase in the density of the composite, which can be regarded as a negative trend. In the case of the developed materials, we decided to apply the concept of single-polymer composites, i.e., materials reinforced with polymer fibers. To improve the compatibility of the material, the matrices of the composite and the reinforcing fibers are prepared from a polymer of the same type or at least from the same group of materials. An example of such a combination is the composites of the All-PP type [46,47], where fibers made of polypropylene homopolymer PP reinforce the matrix made of low melting PP copolymer (cPP). A similar concept has been applied to materials based on thermoplastic polyesters in srPET-type composites, where there is a combination of poly (ethylene terephthalate)-PET homopolymer fibers and a low-temperature PET copolymer (LPET) [48,49,50]. The srLCP type prepreg made on the basis of polyesters was used in the presented research, where reinforcements for the PET copolymer are fibers based on the LCP copolymer (liquid crystalline polymer). LCP polymers in the form of fibers, most commonly found under the trade name Vectran, are a copolymer of 4-hydroxybenzoic acid and 6-hydroxynaphthalene-2-carboxylic acid. Their properties resemble other super-strength fibers such as Kevlar (aramid fibers) or Dyneema/Spectra (polyethylene fibers). They are often used in the production of anti-ballistic fabrics [51,52,53]. In the case of materials developed as part of the discussed work, the key aspect for srLCP composites was their high impact resistance and low density, similarly to the matrix polymer’s density.

An essential aspect of using thermoplastic polyesters as a composite matrix, in this case, is the ability to create permanently welded joints. After the glass transition temperature is exceeded, the mobility of the polymer chains enables the diffusion process to take place; in the case of other thermoplastic materials (polyolefins and polyamides), it is necessary to melt the bonded layer. The high weldability of materials such as PET, GPET, or PLA copolymers has already been used in previous research on overmolding composite prepregs [35,37,54]. The permanent connection of both components of the hybrid part took place without the need to use dedicated heating systems for the joined surface. In the discussed case, we used an analogous material system based on GPET copolymers.

The aim of the presented works was to test the concept of applying the reinforcement for FDM-printed models using the compression molding technique. For comparative purposes, samples made with the developed compression molding technique were compared with the specimens made with the standard FDM technique. The series of material tests included DSC and TGA measurements, the primary purpose of which was the overall assessment of thermal characteristics and phase changes for printed filaments and srLCP prepregs. DMTA thermomechanical analysis and heat deflection temperature-HDT tests were aimed at assessing changes in the stiffness of the obtained samples and determining the possible range of applications. Mechanical tests and, in particular, impact resistance measurements were performed to indicate whether the developed method has an application potential.

## 2. Experimental Section

### 2.1. Materials

During the study, two types of FDM filaments were used to prepare the sandwich’s core structure. The first material was unmodified GPET resin, while the second filament type was carbon fiber reinforced GPET (GPET/CF); according to the producer characteristic, the CF content was 15%. Both materials were supplied by the company Print-Me (Gorzow Wlkp, Poland). The laminate sheets used during this study were produced from the GPET-based hybrid fabric reinforced with high strength liquid crystal polymer (LCP) fibers. The fabric was supplied by Comfil ApS company (Gjern, Denmark); for the purpose of this article, composite sheets are designated as srLCP composite/prepreg.

### 2.2. Sample Preparation

In order to prepare the reinforced laminates, the hybrid fabric (GPET/LCP) was shaped into a thin sheet by a compression molding technique. The process was conducted with the use of a hydraulic press equipped with heating plates, press model Remiplast 7T (Remiplast, Czerwonak, Poland). The forming plates were heated to 200 °C, and after temperature stabilization, the fabric was placed between the plates. A necessary pressure of 70 bar was applied after immediately. To prevent the composite from sticking to the metal parts of the press, the fabric was placed between the pieces of the teflon film. Sheets were molded for 3 min, while after that time, consolidated laminates were placed between two steel plates for cooling. The cooling procedure was performed using another hydraulic press; the initial temperature of the steel plates was around 20 °C, while the cooling time was 5 min.

The core structure of the laminate was prepared by the FDM method. In the case of unreinforced and CF-filled prints, we used the Prusa MK3 printer (PrusaResearch, Praha, Czech Republic). All samples were printed using a 0.8 mm nozzle to avoid clogging the reinforced material. The printing speed for pure GPET was set to 80 mm/s for the infill structure, while shell layers were printed at 40 mm/s. The GPET/CF printing infill and shell speed were reduced to 40 mm/s and 20 mm/s, respectively. For the study, we use the rectilinear type of the infill pattern. The main factor in selecting this geometry is high printing speeds, which are crucial in the case of manufacturing large-size structures. The outer outline of the print was made with a single layer of the shell.

The nozzle’s temperature was set to 250 °C, while the bed temperature was set at 80 °C. Thermal parameters were unchanged for all samples. For the purpose of the experiment, four types of rectangular plates were printed, and each type was distinguished by a different infill density: 10%, 20%, 30%, and 100%. The plate dimensions of 150 mm × 150 mm × 4 mm were constant. The plates were printed without the top and bottom layers of the shell so that the infill structure was visible. In order to compare the properties, reference samples were also prepared. For flexural and impact test performances, we prepared the standard ISO 178 bars with dimensions of 80 mm × 10 mm × 4 mm.

**Figure 1:**
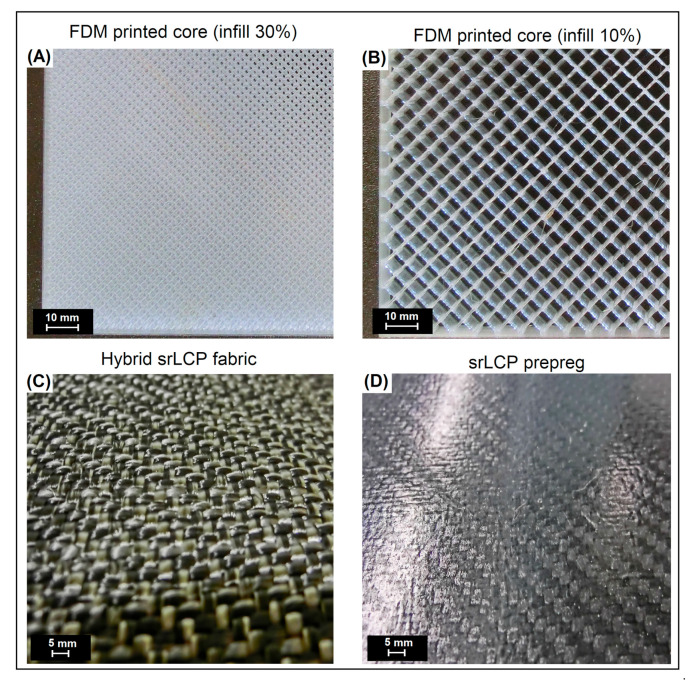
The appearance of the FDM-printed core structures: (**A**) 30% infill density and (**B**) 10% infill density; (**C**) the surface of the hybrid/commingled fabric; and (**D**) compression molded srLCP prepreg.

Sandwich sample preparation was preceded by preliminary tests, where different consolidation temperatures were tested. The initial samples were prepared using previously printed GPET plates with an infill density of 10%. The core plate was fixed between two srLCP sheets and placed between the heated plates of the hydraulic press. Three temperatures were initially tested: 100 °C, 120 °C, and 140 °C. Higher temperatures resulted in remelting materials. Mechanical tests showed the best properties for samples prepared at 140 °C, and that temperature was used to prepare the rest of the specimens. Ready-made samples in the form of sandwich-structured plates were cut into bars in order to conduct mechanical tests. Some pictures presenting the appearance of sandwich structure components can be observed in Figure 1.

### 2.3. Materials Characterization

The sandwich samples as well as the reference specimens were subjected to a detailed analysis of the mechanical properties. At first, the flexural test was conducted. The measurement was performed according to ISO 178 standard using a Zwick/Roell Z010 universal testing machine (Zwick/Roell GmbH, Uilm, Germany), with a load cell capacity of 10 kN. Since the thickness of all samples was around 4 mm, the span distance was set to 64 mm. The impact resistance was measured using the Charpy method according to ISO 179 standard. We used Zwick/Roell HIT25 machine (Zwick/Roell GmbH, Ulm, Germany) equipped with instrumentalized 5 J pendulum. Tests were performed using standard notched samples. The second type of impact resistance measurement was the failing weight test. For this purpose, we used the tubular impact tester type SP1890 (TQC Sheen, Molenbban, Netherlands) designed for testing the impact resistance of coatings. In our case, the device was used to assess the composite behavior during perforation. Before the test, a 50 mm × 50 mm composite plate was mounted on the bottom of the apparatus tube. We used a spherical penetrator with a diameter of 20 mm and 1 kg weight. The tests were performed with the impact energy of 5 and 10 N.

In order to evaluate the differences in thermomechanical properties, samples were subjected to DMTA and HDT tests. A dynamic mechanical thermal analysis (DMTA) was conducted using Anton Paar MCR 301 apparatus; viscoelastic properties were measured using torsion modes. The testing temperature range varied from 25 to 130 °C, while the heating rate was set to 2 °C/min. The strain amplitude was set to 0.01%, while the frequency was 1 Hz. Heat Deflection Temperature measurements were conducted using oil bath HDT/Vicat apparatus, type RV-300C (Testlab, Warsaw, Poland). The test load was 1.8 MPa, while the heating rate was 2 °C/min.

Other thermal properties were measured using DSC and TGA methods. Differential scanning calorimetry (DSC) was used to compare the phase transitions of pure GPET, GPET/CF, and srLCP composites. Tests were conducted using Phoenix DSC 204 machine (Netzsch GmbH, Selb, Germany). Filament material tests were performed from 30 to 250 °C, while the maximum temperature for srLCP composite was 350 °C. The heating/cooling rate was set to 10 °C/min. Samples were prepared by cutting small ≈ 5 mg samples; during the measurement, specimens were placed inside the aluminum crucibles. We also used the protective atmosphere of nitrogen. Thermogravimetric (TGA) tests were conducted with the use of Libra TG 209 (Netzsch GmbH, Selb, Germany). Measurements were performed from 30 to 900 °C, with a heating rate of 10 °C/min. Similarly to the DSC method, TGA tests were conducted under a nitrogen atmosphere. Specimens were prepared by cutting small ≈ 10 mg samples of material, which were placed inside the ceramic crucible.

## 3. Results and Discussion

### 3.1. Thermal Properties-TGA and DSC Thermal Analysis

**Figure 2:**
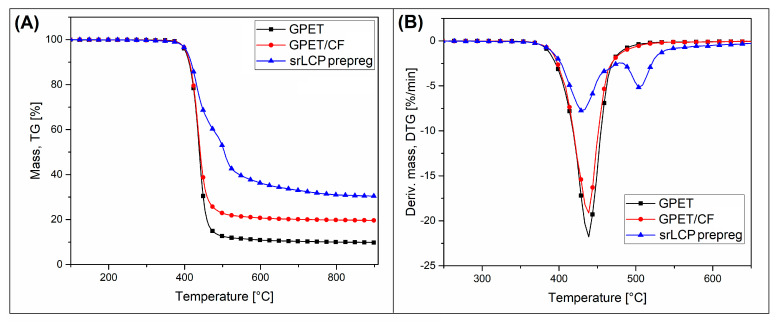
TGA analysis thermograms of the basic materials: GPET and GPET/CF filaments and LCP composite. (**A**) TG mass loss plot and (**B**) DTG derivative mass loss plot.

The results of the measurements made by the TGA analysis method were used to indicate the range of thermal stability of the base materials and to confirm the content of carbon fibers in the structure of the GPET/CF composite. The TG and DTG plots for GPET, GPET/CF, and srLCP composite samples are presented in Figure 2. Up to 400 °C, the mass-loss TG plots for all three materials are similar. As shown by direct readings, 5% weight loss is in the range of 400–405 °C for all samples. This range corresponds to the onset of the GPET matrix’s degradation process. Interestingly, the addition of CF does not change the composite filament properties, which is probably related to a small amount of the fibers in the structure; the same conclusion applies to the composite with LCP/Vectran fibers. The first stage of the decomposition process can also be seen on the DTG plots, where for all samples, the first peak of the plots can be distinguished at 440 °C for all examined materials. The height of the peak is almost equal for pure GPET and GPET/CF samples, while for the srLCP composite, its intensity was reduced. The reason for that is the lower content of the GPET resin for this type of composite at around 50 wt%. The single-step degradation process for filament materials finished at about 500 °C.

Further heating reveals the presence of residue char, whereas for pure GPET resin, the mass at 900 °C was around 10%, while for GPET/CF composites, it was around 19%. Therefore, it can be assumed that the difference of around 10% is the real carbon-fiber content, which is lower than the manufacturer’s indications (15%). Unlike the single-step thermal degradation of filament materials, the srLCP composite decomposition process reveals two visible stages. The first one, as already mentioned, concerns the GPET matrix degradation at 400 °C, while the second one is related to the presence of LCP fibers. Interestingly, the high value of the DTG peak at around 510 °C indicates that the thermal resistance of this kind of polyester is higher than the standard ones such as PET, PBT, or PLA. Moreover, 30% of the residue char content at 900 °C is even higher than for CF-filled GPET materials, suggesting the carbonization phenomenon’s high intensity. The high thermal stability of LCP fibers was already confirmed by many studies [55,56,57]; in most cases, the authors indicate that the decomposition process starts below 500 °C, in which the aromatic compounds are beginning to evolve. The final DTG peak at 510 °C is related to the decomposition of phenolic, aryl ester, and ketone groups.

**Figure 3:**
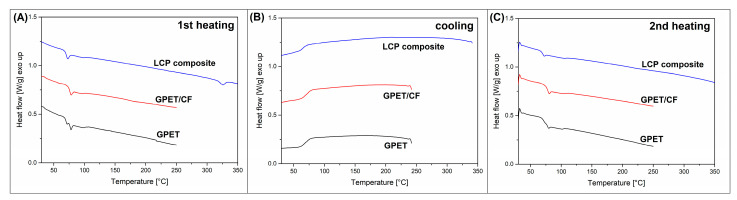
DSC thermograms for pure GPET, GPET/CF, and srLCP composite: (**A**) 1st heating, (**B**) cooling, and (**C**) 2nd heating stage.

The changes in thermal properties analyzed in TGA measurements provide some ideas about the behavior of materials at extremely high temperatures; however, the DSC analysis allows for more detailed analyses in the temperature range of application and processing of the discussed materials. The results of first heating/cooling/second heating DSC measurements are presented in Figure 3, in which the thermograms of GPET, GPET/PC, and srLCP composite material are shown. For both filament materials, the fully amorphous structure reveals no visible melting peaks; however, the presence of the glass transition phenomenon can be observed at around 75 °C. Interestingly for pure GPET, two separate steps can be detected, which might suggest the presence of two types of polymers or bimodal structure of the materials; however, usually, for highly amorphous polymers, it indicates the presence of materials stress. For the srLCP composite, the maximum heating temperature increased to 350 °C in order to detect the melting of the fiber structure, which was confirmed at 326 °C. Similarly to filament materials, the glass transition of the GPET matrix was detected at around 75 °C. The cooling thermograms did not reveal any difference between materials except for the presence of the GPET glass transition. The second heating step reveals that the crystalline structure of the LCP (Vectran) fibers was not formed, which confirmed that, similarly for many other polyester resins, the growth of the crystalline phase is highly time and temperature dependent. The cooling rate of the DSC measurement (10 °C/min) was too high for the formation of the crystalline phase. The appearance of the second heating scan for the filament materials did not change. The only remarkable change concerns the presence of a single glass transition for pure GPET materials, which confirmed that the double T_g_ step at first heating was the result of the material’s internal stress.

Considering the results of TGA and DSC thermal analyses, it is worth noting that the addition of carbon fibers and LCP does not significantly affect the behavior of the GPET matrix. It allows it to assume that the bonding process of sandwich structures will depend mainly on the temperature’s conditions.

### 3.2. Thermomechanical Properties HDT and DMTA

One of the main advantages of using thermoplastic polyesters for the FDM process is their relatively low glass transition and low crystallinity levels. Both features are unfavorable considering the material heat resistance, while the maximum operating temperature is limited. For GPET-based materials, in which the glass transition region ranges from around 75 to 85 °C, the HDT temperature is even lower as confirmed by other studies [58,59,60]. Improvements in the thermomechanical properties of amorphous polymers are possible only to a minimal extent; therefore, using carbon fibers in one of the tested filaments does not significantly impact the heat resistance results such as HDT (see Table 1). The initial HDT tests were performed for all prepared samples, including both FDM-printed and reinforced sandwiches. The initial heat resistance of 100% infill GPET and GPET/CF was very similar at 71.5 °C and 73.1 °C, respectively. The HDT results for the other samples were slightly lower; however, the heat resistance for the 10% infill samples was only 2–3 °C lower, which cannot be considered a significant reduction.

Interestingly, the thermal resistance of sandwich samples is the lowest. The difference between unreinforced samples and srLCP prepreg-reinforced samples ranges from 3 to 6 °C. The decrease was observed for all of the prepared specimens, which means that the observed behavior was a constant trend suggesting that the addition of the reinforcing sheet results in a visible decrease in the sample’s heat resistance.

**Figure 4:**
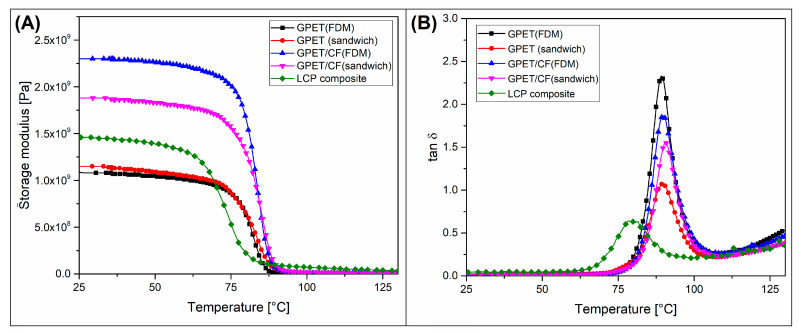
Storage modulus (**A**) and tan δ (**B**) thermograms for different types of samples.

The DMTA analysis was performed for all of the prepared 100% infill specimens, and the viscoelastic properties of the reference srLCP sheet sample were evaluated. As observed in Figure 4, the difference in storage modulus between the printed GPET sample and GPET/LCP sandwich was very small, and the stiffness of the reinforced sample was only slightly higher. Interestingly, for GPET/CF materials, the storage modulus of the FDM-printed models was significantly higher than thermogram values for a sandwich specimen. The main reason for this opposite trend is the presence of the prepreg sheet within the sandwich structure. The storage modulus values of the srLCP composite were slightly higher than the pure GPET materials but significantly lower than the stiffness of the GPET/CF sample.

Consequently, the reinforcement efficiency for GPET-based sandwiches was very low, while the GPET/CF-based sandwich’s stiffness was strongly reduced. Moreover, the glass transition region for srLCP sheet materials was reduced, which can be observed when analyzing the tan δ plot. The thermogram peak for the srLCP sample was recorded at around 80 °C, while for the printed specimens, it was close to 90 °C. The reason for that temperature shift was the use of a different polyester resin to prepare the srLCP composite. The used resin is certainly an amorphous form of polyethylene terephthalate, most possibly a GPET copolymer. However, due to the use as a matrix for LCP fibers, this material must be characterized by low viscosity during the compression molding process of the hybrid fabric, i.e., around 200–250 °C. Most of the standard varieties of GPET resin are characterized by high viscosities in this temperature range, which is also an advantage in the 3D-printing process. In summary, the thermomechanical properties investigation revealed that the thermal resistance of the prepared composite structures was not improved; in the case of sandwich structures, the HDT was even slightly lower than for unmodified GPET. Nevertheless, the fact that the improvement of thermomechanical properties was not the expected goal of the conducted works. The above studies state that, to obtain a broader range of applications, it would be worth considering the use of blends with the addition of more heat-resistant PC or ABS as a matrix. The use of fibrous fillers does not change the HDT value, which is indirectly related to the relatively low content of the reinforcing fibers, usually for FDM filaments at the maximum level of about 20%.

### 3.3. Mechanical Properties-Flexural Tests and Charpy Impact Resistance Measurements

**Figure 5:**
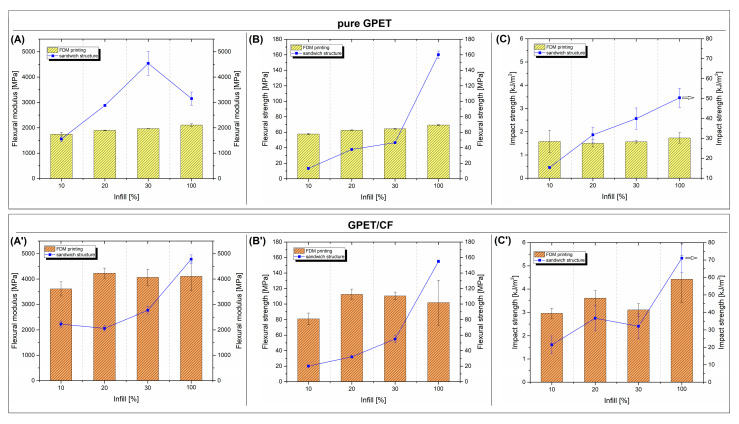
Mechanical properties of FDM-printed samples and compression molded sandwich structures: (**A**,**A’**) flexural modulus, (**B**,**B’**) flexural strength, and (**C**,**C’**) Charpy impact strength. The top plots present the results for pure GPET samples, while the bottom plots are for GPET/CF materials.

**Figure 6:**
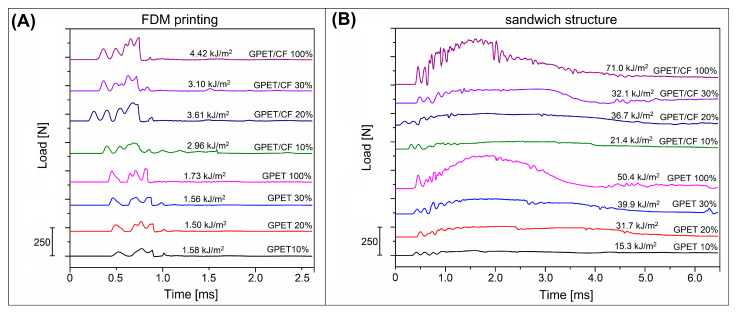
The load versus time plots recorded during the Charpy impact test: (**A**) for FDM-printed samples (**B**) and compression molded sandwich samples.

**Figure 7:**
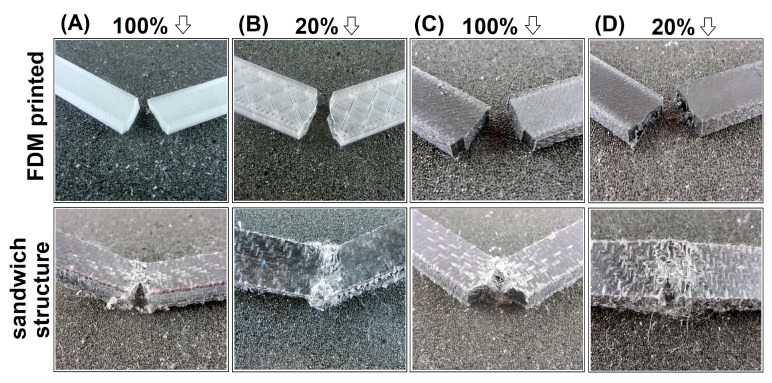
The appearance of the notched samples after the test. The picture at the top presents the FDM-printed specimen, while sandwich samples are at the bottom: (**A**) pure GPET–100% infill, (**B**) pure GPET–20% infill, (**C**) GPET/CF–100% infill, and (**D**) GPET/CF–20% infill.

The mechanical properties of the prepared samples have been evaluated using static flexural tests and Charpy impact measurements. The results in the form of flexural modulus, flexural strength, and impact strength plots are presented in Figure 5. The plots illustrate the comparison between the FDM-printed samples and compression-molded sandwich sheets. Additional pictures from Figure 6 show the load vs. time plots recorded during the Charpy impact tests. The images from Figure 7 collect the photographs of the specimens after the test.

According to the obtained results, the mechanical properties of the pure GPET printed samples are only slightly influenced by the infill’s density. The influence of the infill density is mainly negligible. The increase between the 10% and 100% infill samples for the flexural modulus and strength was only around 15%, and for impact strengths, the increase was approximately 10%. Visible improvements in mechanical properties were observed for samples with CF reinforcements; however, the changes between the samples with different infill densities are again insignificant.

The changes observed for the sandwich samples are more significant. The flexural modulus of the samples made from the pure GPET core systematically increased, reaching a maximum of around 4.5 GPa for a 30% infill sandwich. While for 100% infill samples, the stiffness was reduced to 3.1 GPa. This difference is quite surprising as the best-expected results should be for samples with full fillings. The reduction in stiffness can be caused by a large change in the geometry of the infill structure between 30% and 100% for solid samples (100% infill) in which the individual layers of the specimen are connected at an angle of 90 degrees. In contrast, for partially infill samples, the layers are stacked on top of each other with no angle deviations. However, this theory would have to be reflected for materials based on GPET/CF filaments, which is not the case; thus, it is probable that other factors are more critical for this particular example. Based on current research, it is quite difficult to precisely analyze this type of behavior.

For GPET/CF-based samples, the increase in the infill’s density also improved the stiffness; however, the results for models up to 30% infill showed a slight increase of about 20%. The use of prepregs in solid samples (100% infill) showed a significant improvement, in which the modulus value doubled compared to the initial 10% infill sample. Only for the 100% infill hybrid sandwich was the stiffness higher than that of the printed models. At the same time, the flexural modulus for the rest of the specimens was significantly lower for sandwich materials. The reason for that behavior is the relatively low stiffness in the transverse direction for the srLCP prepreg. In this case, the addition of the prepreg reduces the amount of CF fibers, which significantly increases the modulus’s value. The relationship regarding flexural strength is identical for GPET and GPET/CF samples; in both cases, an increase in the infill density causes an increase in strength, but the obtained flexural strength was higher only for the 100% infill sandwich samples than for the printed materials. In contrast to the results of the static tests, the results of the impact resistance measurements clearly show the favorable properties of the developed sandwich system.

The impact strength for all samples increases by a minimum of one magnitude. Even for 10% infill GPET samples, the impact strength values increased from 1.6 kJ/m^2^ to 15.3 kJ/m^2^, while for the solid specimen, the strength reached 50 kJ/m^2^. The increase was even higher for GPET/CF-based samples, while for the initial 10% infill sandwich, the resulting strength was 21.4 kJ/m^2^, while the impact resistance of the solid sample was the highest of all materials and reached 71 kJ/m^2^. The results of the impact tests are supplemented by the load versus time plots recorded during the sample’s fracture. As observed in Figure 6A, despite some differences in impact strength values, the plots for all printed samples look very similar and clearly indicate the brittle nature of the crack in the samples. The average time of the full fracture was below 1 ms. The completely different nature of the cracking process is visible for the sandwich sample Figure 6B in which none of the samples with the addition of the srLCP sheet were fractured. Interestingly, during the test, the prepreg surface did not detach from the printed core (see Figure 7), which proves good adhesion at the border of the joined parts.

The most advanced composite solutions are based on carbon fibers or hybrid fiber systems and epoxy resin as the matrix compound [52,61,62], while for sandwich structures, part of the manufactured structures can be made from metal (steel and aluminum) in the form of a thin sheet or foam [63,64,65,66]. The obtained results indicate that this kind of sandwich materials cannot be used in high-demand applications, such as the aeronautic industry [67,68] or automotive and sports products [69,70,71]. The results of static measurements indicate no spectacular improvement in the mechanical characteristics of the developed hybrid system. The significant improvement concerns impact resistance. In the discussed context, however, it seems reasonable to carry out further works aimed at improving the stiffness of the tested system including, firstly, optimizing the infill structure or/and by changing the reinforcing sheet’s composition.

**Figure 8:**
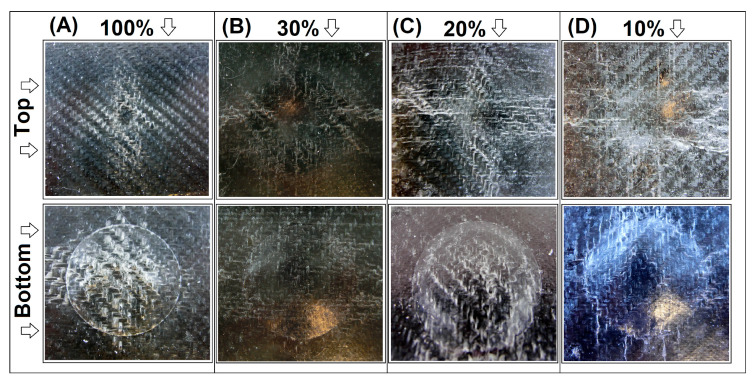
The appearance of the top and bottom surface of the sandwich sample after the drop weight test. Pure GPET samples: (**A**) 100% infill, (**B**) 30% infill, (**C**) 20% infill, and (**D**) 10% infill.

**Figure 9:**
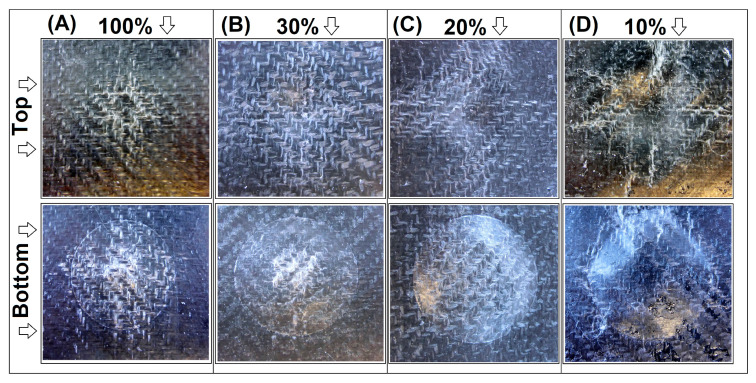
The appearance of the top and bottom surface of the sandwich sample after the drop weight test. GPET/CF samples: (**A**) 100% infill, (**B**) 30% infill, (**C**) 20% infill, and (**D**) 10% infill.

Mechanical tests are supplemented with the puncture tests performed using the falling weight method. For all tested sandwich panels, the structure could not be fully pierced, which confirms the high efficiency of the used solution. The pictures in Figure 8 and Figure 9 present the appearance of the sample after measurement with the highest available load of 10 N, separately for pure GPET samples and GPET/CF-based materials. For the samples prepared with the standard FDM technique (without the srLCP prepregs), all specimens were fractured completely, even at the impact energy of 5 N, which is why we did not compare them in this analysis.

For the GPET-based structure of the 100% infill sample, the surface of the composite was only slightly deformed, which can be observed in detail at the specimen’s top surface. Only a small indentation is visible at the point of the impact. The characteristic white streaks spreading from the point of impact, in accordance with the orientation of the fibers at 0° and 90°, indicate the delamination of the fibers in the whitening zone. The underside of the sample shows much more significant damage to the structure. The whitened surface is still oriented in the direction of the fibers in the fabric, while the area is much larger. This damage suggests that the impact energy was absorbed mainly by the bottom part of the composite. In the case of other sample types, it is clear that the infill’s density affects the penetration’s depth. Interestingly, the lower surface of the sandwich samples is characterized by significant convexity, while the prepreg surface’s whitening is not as significant as in the 100% infill sample. Such behavior suggests that the composite core structure has accumulated a significant part of the impact energy as opposed to a full infill sample where most of the energy was transferred to the srLCP prepreg layer.

In the case of GPET/CF-based materials, the appearance of the tested specimens was very close to pure GPET samples. The sample with full infills turned out to be the least deformed, while the decreasing content of the infill material led to more significant deformations on the sandwich’s surface. However, unlike the other samples, none of the prepregs were punctured; thus, the srLCP composite layer fulfilled its function in all tested samples.

It is quite clear that for this type of puncture test, it is only possible to compare the effects caused by the striking dart. Some other studies used instrumentalized equipment in the form of drop-weight hammers also for LCP fiber-reinforced composites [52,72]. However, the main tested materials are still solid thermoset-based laminates. It is difficult to find similar tests on the presented hybrid structures, while some interesting research was already conducted on the subject of FDM-printed sandwich materials. This type of publication includes research on the analysis of sandwich structures produced using different variants of the infill structure, which was presented by Yazdani Servestani et al. [38]. Other research presented by Jhou et al. [73] deals with similar concepts, using more advanced lattice structures. In both cases, the research concept was focused on developing a faithful mathematical model enabling practical simulation analyses of the developed materials. The results of the drop weight tests were focused mainly on the deformation analysis and matching its value to the simulation model. Dou et al. [74] studied what certain contexts may constitute a valuable reference to the values obtained for the prepared hybrid structures. The discussed study included the evaluation of the strength of a PLA-based honeycomb structure reinforced with continuous carbon fiber. The concept of material production to some extent resembles the presented method of producing srLCP hybrid structures, while in both cases, the starting point was the infill structure produced by the FDM technique. For the cited study, the drop-weight tests for continuous carbon fiber-reinforced structures revealed a large improvement in maximum force of the impact and energy absorption. Compared to the unreinforced sample, the force increased from 512 N to 1353 N, while energy increased from 173 J to 537 J. This kind of difference indicates a strong impact resistance enhancement, similarly to the discussed studies in which Charpy tests indicated a minimum tenfold increase in the impact toughness for hybrid GPET/srLCP sandwich structures.

## 4. Conclusions

The main goal of the work was an attempt to use composites based on LCP fibers to strengthen the FDM-printed parts. The presented tests are an introduction to further work for obtaining more complex products with spatial geometries. Sandwich structures prepared during this research are characterized by high impact strength, which confirms the effectiveness of the applied concept. Although the results indicate that the best results are obtained for samples with a 100% infill density, even for sandwich samples with a 10% degree of filling, the impact strength increases up to ten times. This is confirmed by the excellent results of the penetration tests. Thermomechanical properties measurements showed no changes in thermal resistance, which for most GPET-based materials do not exceed 70 °C. It is worth mentioning that this copolymer’s low glass transition temperature facilitates the formation of a permanent bond between the reinforcing srLCP prepreg and the FDM-printed core.

## Figures and Tables

**Table 1:** Results of the HDT measurements for FDM-printed samples and sandwich specimens.

Infill Density	Pure GPET		GPET/CF	
	FDM Printed	Sandwich Structure	FDM Printed	Sandwich Structure
10%	68.4 (±0.9)	66.9 (±0.3)	72.1 (±0.2)	65.1 (±2.8)
20%	69.8 (±1.3)	65.5 (±4.1)	73.7 (±0.5)	67.5 (±3.3)
30%	72.1 (±1.3)	66.4 (±3.2)	74.6 (±0.8)	68.9 (±2.1)
100%	71.5 (±0.3)	68.2 (±1.3)	73.1 (±0.2)	69.5 (±2.3)

## Data Availability

Not applicable.
